# US Geographical Variation in Rates of Shoulder and Knee Arthroscopy and Association With Orthopedist Density

**DOI:** 10.1001/jamanetworkopen.2019.17315

**Published:** 2019-12-11

**Authors:** Nitin B. Jain, Emily Peterson, Gregory D. Ayers, Amos Song, John E. Kuhn

**Affiliations:** 1 Department of Physical Medicine and Rehabilitation, Vanderbilt University School of Medicine, Nashville, Tennessee; 2 Department of Orthopaedics and Rehabilitation, Vanderbilt University School of Medicine, Nashville, Tennessee; 3 Department of Biostatistics, University of Massachusetts, Amherst; 4 Department of Biostatistics, Vanderbilt University School of Medicine, Nashville, Tennessee

## Abstract

**Importance:**

Although rates of arthroscopy have substantially increased, recent data question its comparative effectiveness.

**Objectives:**

To assess time trends and geographical variations among several US states in arthroscopy rates and to assess the association of orthopedist density with arthroscopy rates.

**Design, Setting, and Participants:**

In this cross-sectional study, procedure rates were calculated for knee arthroscopy, shoulder arthroscopy, and arthroscopic rotator cuff repair using data from the State Ambulatory Surgery and Services Databases for 2006 to 2016 (as available) for the states of California, Colorado, Florida, Iowa, Kentucky, Maryland, Maine, Michigan, Minnesota, North Carolina, Nebraska, New Jersey, Nevada, New York, Oregon, Utah, Vermont, and Wisconsin. Data were analyzed from June 2017 to October 2019.

**Main Outcomes and Measures:**

Rates of knee arthroscopy, shoulder arthroscopy, and arthroscopic rotator cuff repair.

**Results:**

The combined data sets included 4 856 385 records with 2 530 840 female patients (47%); mean (SD) patient age was 49.13 (16.34) years. Rates per 100 000 persons showed large geographical variations for knee arthroscopy (from 63.31 [95% CI, 5.92-198.95] to 721.72 [95% CI, 633.41-806.20]), shoulder arthroscopy (from 53.02 [95% CI, 2.80-164.36] to 438.25 [95% CI, 399.00-476.78]), and arthroscopic rotator cuff repair (from 11.94 [95% CI, 1.30-56.98] to 185.35 [95% CI, 143.84-226.20]) across US states and years. There were significant downward time trends in knee arthroscopy rates in California, Florida, Iowa, Maryland, Michigan, Nebraska, and New Jersey and upward trends for arthroscopic rotator cuff repair in Colorado, Florida, Kentucky, Maine, and North Carolina. Orthopedist density was not associated with knee arthroscopy rates (slope = 3.07; 95% CI, −9.88 to 16.03; *P* = .54), shoulder arthroscopy rates (slope = 2.74; 95% CI, −6.53 to 12.01; *P* = .47), or rates of arthroscopic rotator cuff repair (slope = 1.15; 95% CI, −2.77 to 5.05; *P* = .49).

**Conclusions and Relevance:**

There is large geographical variation in arthroscopy rates despite the questionable comparative effectiveness of these procedures. The reasons for increasing rates of rotator cuff surgery should be further examined.

## Key Points

**Question:**

Is there geographical variation in arthroscopy rates among US states and are these rates associated with orthopedist density?

**Findings:**

This cross-sectional study found that rates per 100 000 persons showed large geographical variations for knee arthroscopy (63.31-721.72), shoulder arthroscopy (53.02-438.25), and arthroscopic rotator cuff repair (11.94-185.35) across US states and years. Orthopedist density was not significantly associated with knee arthroscopy, shoulder arthroscopy, and arthroscopic rotator cuff repair.

**Meaning:**

These findings suggest that there is large geographical variation in arthroscopy rates despite the questionable comparative effectiveness of these procedures.

## Teaser

This cross-sectional study estimates time trends and geographical variations in rates of arthroscopic surgery among US states and investigates whether rates of arthroscopy are associated with geographic density of orthopedists.

## Introduction

Knee and shoulder concerns were 2 of the top 15 reasons for patients to seek ambulatory care during physician office visits in 2015 (28.9 million estimated visits combined).^1^ An estimated 984 607 knee arthroscopic procedures and 529 689 rotator cuff repairs and shoulder arthroscopic procedures were performed on an ambulatory basis in 2006.^2,3^ In the last 10 to 15 years, evidence has suggested that there were no significant differences between patients undergoing arthroscopic surgery vs sham surgery or nonoperative treatment for knee disorders such as osteoarthritis and meniscal tears^4,5,6,7^ and for shoulder impingement.^8^ Although national estimates are available for knee and shoulder arthroscopy from the 2000s,^2,9,10^ there is likely variation among states^11^ due to differences in practice patterns, patient preferences, and health insurance and reimbursement environments. Contemporary arthroscopy estimates for US states are unavailable from reliable data sources.

The concept of elasticity has been applied to surgical services such that with an increase in surgeon density, an increased demand for surgery is expected.^12,13^ To our knowledge, the association of orthopedic surgeon density with knee and shoulder arthroscopy rates has not been studied, and the variation of this association in recent years across states is unknown.

We used databases for several states across the United States to assess trends and geographical variation in rates of knee arthroscopy, shoulder arthroscopy, and arthroscopic rotator cuff repair from 2006 to 2016. We assessed the association of orthopedic surgeon density with knee arthroscopy, shoulder arthroscopy, and arthroscopic rotator cuff repair.

## Methods

### Patient Population

Arthroscopy is performed almost exclusively on an ambulatory basis in the United States.^9^ In this cross-sectional study, we used the State Ambulatory Surgery and Services Databases (SASD)^14^ for the years 2006 through 2016 (as available) for California (CA), Colorado (CO), Florida (FL), Iowa (IA), Kentucky (KY), Maryland (MD), Maine (ME), Michigan (MI), Minnesota (MN), North Carolina (NC), Nebraska (NE), New Jersey (NJ), Nevada (NV), New York (NY), Oregon (OR), Utah (UT), Vermont (VT), and Wisconsin (WI). The SASD is part of the Healthcare Cost and Utilization Project sponsored by the Agency for Healthcare Research and Quality.^15^ The SASD has encounter-level data for ambulatory surgical procedures from hospital-owned facilities for all states and non–hospital-owned facilities in some states (CA, FL, KY, NV, NY, NC, OR, UT, and WI). Healthcare Cost and Utilization Project databases have been extensively used to estimate population-based estimates and trends for a variety of medical conditions.^16,17,18^ Further details about the database, comparison with American Hospital Association Annual Survey data, and validation can be found elsewhere.^14,15,19^

This study was granted a waiver by the institutional review board at Vanderbilt University owing to the use of deidentified data. Reporting follows the Strengthening the Reporting of Observational Studies in Epidemiology (STROBE) reporting guideline. Data were analyzed from June 2017 to October 2019.

### Arthroscopy Procedures

The SASD has information on *Current Procedural Terminology* (*CPT*) codes that were developed by the American Medical Association.^20^ Both primary and secondary procedure codes were available for each patient record. Primary and secondary *CPT* codes (up to 10 codes for each encounter) for knee arthroscopy, shoulder arthroscopy, and arthroscopic rotator cuff repair were used to extract patient records of interest (eTable in the Supplement). The *CPT* codes for shoulder arthroscopy included those for arthroscopic rotator cuff repair in addition to other procedure codes as outlined in the eTable in the Supplement.

### Orthopedic Surgeon Density

The American Academy of Orthopaedic Surgeons (AAOS) practice reports^21^ provided the density of orthopedic surgeons by state and year. The report included “practicing orthopedists in the AAOS membership categories of candidate member practitioners (CMP’s), CMP fellow applicants, applicants for fellowship, fellows, non-member practitioners, and emeritus fellows”^21^ to calculate orthopedic surgeon density. These reports were available for the years 2006, 2008, 2010, 2012, 2014, and 2016.

### Statistical Analysis

Observed state-year rates of knee arthroscopy, shoulder arthroscopy, and arthroscopic rotator cuff repair were calculated from 2006 to 2016, as available, for US states included in SASD. The Mann-Kendall test was performed to identify statistically significant state-specific time trends that were monotonically increasing or decreasing trends and could not be considered random realizations.^22^

Population standardized estimates stratified by state, year, and age and sex reported by the US Census Bureau were used to calculate procedure rates per 100 000 persons.^23^ To describe the uncertainty in observed state-specific time trends, 95% confidence intervals were calculated, using estimated sampling error given by a truncated normally distributed data assumption.

Standardized estimates were also calculated by state, year, and household income quartile using information reported on median household income of patient county residence and median household income population counts. The SASD databases separate household income into the following quartiles: (1) less than $35 000, (2) $35 000 to $49 999, (3) $50 000 to $74 999, and (4) $75 000 or greater. In the absence of information on the income of the individual patient, SASD reports the median household income quartile for the corresponding county of the patient’s residence.

To assess the association between orthopedic surgeon density and changes in procedure rates of knee arthroscopy, shoulder arthroscopy, and rotator cuff surgery, a linear regression was used in which we adjusted for state-specific time trends by including terms for state-specific intercepts and time. Statistical significance was set at 2-tailed *P* < .05. All statistical analyses were performed using R software version 3.5.0 (R Project for Statistical Computing)^24^ by 1 of us (E.P.).

## Results

**Figure 1.**
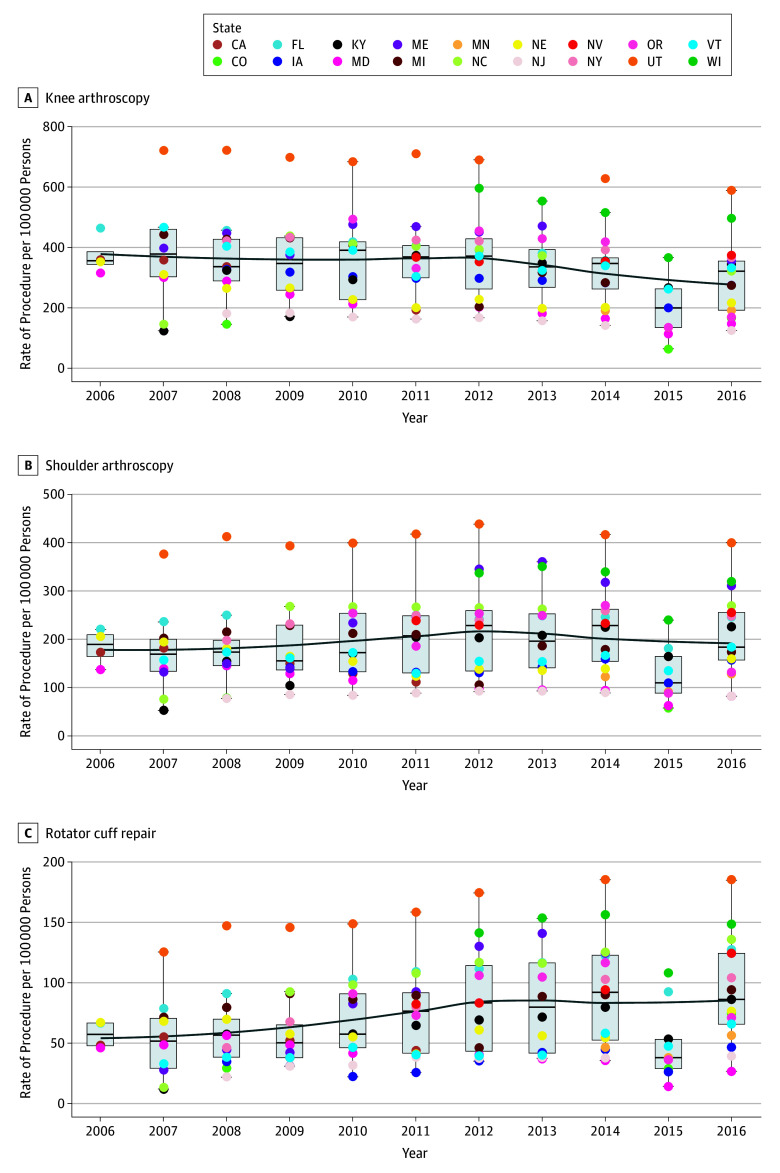
Rates of Ambulatory Knee Arthroscopy, Shoulder Arthroscopy, and Arthroscopic Rotator Cuff Repair in Select US States, 2006-2016 CA indicates California; CO, Colorado; FL, Florida; IA, Iowa; KY, Kentucky; MD, Maryland; ME, Maine; MI, Michigan; MN, Minnesota; NC, North Carolina; NE, Nebraska; NJ, New Jersey; NV, Nevada; NY, New York; OR, Oregon; UT, Utah; VT, Vermont; and WI, Wisconsin.

The combined data sets included 4 856 385 records with 2 530 840 female patients (47%); mean (SD) age was 49.13 (16.34) years. Large geographical variation in rates of knee arthroscopy, shoulder arthroscopy, and arthroscopic rotator cuff repair were observed among states from 2006 to 2016 (Figure 1). Rates per 100 000 persons showed large geographical variations for knee arthroscopy (from 63.31 [95% CI, 5.92-198.95] to 721.72 [95% CI, 633.41-806.20]), shoulder arthroscopy (from 53.02 [95% CI, 2.80-164.36] to 438.25 [95% CI, 399.00-476.78]), and arthroscopic rotator cuff repair (from 11.94 [95% CI, 1.30-56.98] to 185.35 [95% CI, 143.84-226.20]) across US states and years. Utah had the highest procedure rates for most years. In 2007, procedure rates per 100 000 persons in Utah were 721 (95% CI, 633.41-806.19) for knee arthroscopy, 376.06 (95% CI, 335.86-376.06) for shoulder arthroscopy, and 125.45 (95% CI, 82.0-169.48) for rotator cuff repair. In 2014, these rates were 627.61 (95% CI, 539.59-716.68) for knee arthroscopy, 416.5 (95% CI, 376.1-454.54) for shoulder arthroscopy, and 185.32 (95% CI, 144.54-228.16) for rotator cuff repair. A significant downward trend over time in the rate of knee arthroscopy was seen for CA, FL, IA, MD, MI, NE, NJ, NC, UT, and VT (eFigure 1 in the Supplement; *P* for trend < .05 for all states). No state had a significant upward trend in rates of knee arthroscopy. A significant decrease in shoulder arthroscopy rates was seen for MD (*P* for trend = .001) and NE (*P* for trend = .03), and a significant trend of increase in shoulder arthroscopy rates was seen for KY (*P* for trend = .007) and ME (*P* for trend = .02). For rotator cuff repair, a significant monotonic decrease trend was seen for MD (*P* for trend = .002), whereas a significant increase trend was seen for FL, KY, ME, NY, NJ, NC, UT, and VT (*P* for trend < .05 for all). Age and sex–standardized and income-standardized rates of knee arthroscopy, shoulder arthroscopy, and arthroscopic rotator cuff repair are presented in eFigure 2 and eFigure 3 in the Supplement.

**Figure 2.**
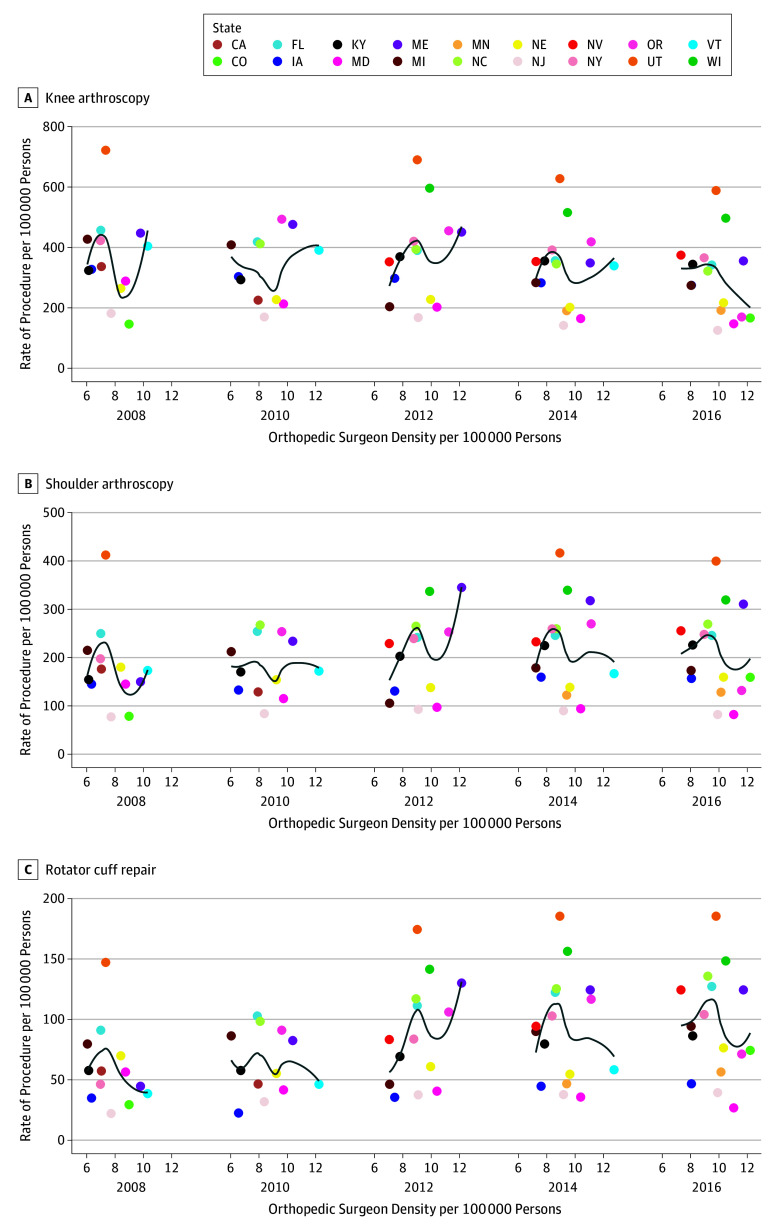
Rates of Knee Arthroscopy, Shoulder Arthroscopy, and Arthroscopic Rotator Cuff Repair in Select US States by Density of Orthopedic Surgeons CA indicates California; CO, Colorado; FL, Florida; IA, Iowa; KY, Kentucky; MD, Maryland; ME, Maine; MI, Michigan; MN, Minnesota; NC, North Carolina; NE, Nebraska; NJ, New Jersey; NV, Nevada; NY, New York; OR, Oregon; UT, Utah; VT, Vermont; and WI, Wisconsin.

Orthopedic surgeon density and procedure rates for knee arthroscopy, shoulder arthroscopy, and arthroscopic rotator cuff repair for various states are represented in Figure 2. After adjusting for time trends and state variations in procedure rates, orthopedic surgeon density was not associated with rates of knee arthroscopy (slope = 3.07; 95% CI, −9.88 to 16.03; *P* = .54), shoulder arthroscopy (slope = 2.74; 95% CI, −6.53 to 12.01; *P* = .47), or arthroscopic rotator cuff repair (slope = 1.15; 95% CI, −2.77 to 5.05; *P* = .49) (eFigure 4 in the Supplement).

## Discussion

There were large geographical variations among US states in the performance of shoulder, rotator cuff, and knee arthroscopic surgery on an ambulatory basis from 2006 to 2016. Most states had a significant decrease in rates of knee arthroscopy. However, rates of arthroscopic rotator cuff repair significantly increased for several states and only 2 states had a decrease. Orthopedic surgeon density was not associated with rates of any of the 3 types of procedure studied after accounting for state-specific time trends.

With the advent of arthroscopy, the rates of soft-tissue shoulder and knee surgical procedures have increased rapidly over time according to data from the 1990s and the 2000s.^3,9,25,26^ However, large randomized clinical trials reported in the last 10 to 15 years provide evidence that arthroscopy does not result in significantly better patient-reported outcomes compared with physical therapy or sham surgery for the knee for osteoarthritis and degenerative meniscal tears.^4,5,6,7^ It is unclear whether arthroscopy rates have been affected by these data. Our study shows that there is substantial geographical variation in arthroscopy rates, as was reported for shoulder arthroplasty and rotator cuff repair using Medicare data from 1992^11^ and for total hip and knee arthroplasty.^27^ Knee arthroscopy rates in several states decreased significantly, and no state had a significant increase in rate of knee arthroscopy over time. However, shoulder arthroscopy rates, and especially rates of arthroscopic rotator cuff repair, have significantly increased in several states over time. This may reflect the availability of evidence from large randomized trials on knee arthroscopy that is lacking for arthroscopic rotator cuff repair. Similar results of decreasing rates were reported using state data from 1998 to 2006^28^ and using American Board of Orthopaedic Surgery data from 1999 to 2009^29^ for knee arthroscopy for osteoarthritis after publication of the trial by Moseley et al^6^ in 2002. Trends in knee arthroscopy seem to follow the data that question its effectiveness vs physical therapy or sham surgery in osteoarthritis and meniscal disorders,^4,5,6,7^ although large pragmatic trials are still lacking in this area. It is not known whether this trend reflects change in practice recommendations of orthopedic surgeons, patent preferences, reimbursement issues that are governed by health insurers, or changing population demographics. Data from our study may be useful for the ongoing policy conversations on bundled payments.

The question of whether a greater availability of orthopedic surgeons is associated with higher rates of arthroscopic procedures is controversial. Vitale et al^11^ reported that rates of shoulder procedures were not significantly associated with orthopedist density. Our study showed that orthopedic surgeon density was not associated with rates of knee and shoulder arthroscopy and arthroscopic rotator cuff repair before and after controlling for state-specific time trends. It is possible that the availability of recent evidence on questionable effectiveness of knee arthroscopy vs physical therapy or sham surgery^4,5,6,7^ has led to this.

### Limitations

This study has some limitations, such as the noninclusion of rotator cuff, meniscal, and anterior or posterior cruciate ligament surgical procedures performed in nonambulatory facility settings and as open procedures. However, since the 2000s, most of these procedures are performed arthroscopically and in ambulatory settings.^9,25,26^ Miscoding of procedures is a possibility, although *CPT* codes are quite accurate^30,31^ because they represent surgical procedures that are used for billing and there is no reason to believe that this coding bias was differential across states and years of our study. The state databases are not nationally representative and should not be used to make national inferences. We were also limited in our ability to only report on states that contributed toward SASD. For the orthopedist density analyses, we do not have data on subspecialization. Hence, density of surgeons performing arthroscopy may be different than the density of orthopedic surgeons, although our data may be more applicable because general orthopedists often perform arthroscopy in community settings. We did not assess the association of multiple covariates that may be associated with state differences in arthroscopy rates, such as state-specific health care coverage policies. The regression analyses assessing covariate associations were for exploratory purposes only and are not comprehensive models for prediction or inference.

## Conclusions

In this cross-sectional study, we present contemporary data on geographical variation in knee, shoulder, and rotator cuff repair arthroscopy rates in US states. There was significant geographical variation in arthroscopy rates, likely representing the role of factors such as variation in practice patterns, health insurance and reimbursement, and the presence of underserved areas and populations in the absence of concrete indications for arthroscopic procedures. A significant downward trend over time for knee arthroscopy rates and a significant upward trend over time for arthroscopic rotator cuff repair rates were seen for several states. Orthopedic surgeon density was not associated with arthroscopy rates.

## Notes

Supplement. **eTable.** CPT Codes Used for Shoulder and Knee Arthroscopy Procedures **eFigure 1.** Trends in Rates of Ambulatory Knee Arthroscopy, Shoulder Arthroscopy, and Arthroscopic Rotator Cuff Repair in Select US States (2006-2016) **eFigure 2.** Age and Sex–Standardized Rates of Arthroscopy in Select US States (2006-2016) **eFigure 3.** Income-Standardized Rates of Arthroscopy in Select US States (2006-2016) **eFigure 4.** Association of Orthopedic Surgeon Density With Rates of Arthroscopic Procedures in Select US States (2006-2016)
