# Error in Figures

**DOI:** 10.1001/jamanetworkopen.2020.0317

**Published:** 2020-02-05

In the Original Investigation titled “US Geographical Variation in Rates of Shoulder and Knee Arthroscopy and Association With Orthopedist Density,”^1^ published December 11, 2019, there were errors in keys of Figure 1 and Figure 2. The color representing Utah was transposed with the color representing Minnesota. This article has been corrected.^1^
