# Supplementary material for: US Geographical Variation in Rates of Shoulder and Knee Arthroscopy and Association With Orthopedist Density
Source: JAMA Netw Open. 2019 Dec 11;2(12):e1917315. doi: 10.1001/jamanetworkopen.2019.17315 (PMC6991208; doi:10.1001/jamanetworkopen.2019.17315)
Supplement: Supplement. — eTable. CPT Codes Used for Shoulder and Knee Arthroscopy Procedures eFigure 1. Trends in Rates of Ambulatory Knee Arthroscopy, Shoulder Arthroscopy, and Arthroscopic Rotator Cuff Repair in Select US States (2006-2016) eFigure 2. Age and Sex–Standardized Rates of Arthroscopy in Select US States (2006-2016) eFigure 3. Income-Standardized Rates of Arthroscopy in Select US States (2006-2016) eFigure 4. Association of Orthopedic Surgeon Density With Rates of Arthroscopic Procedures in Select US States (2006-2016) [file jamanetwopen-e1917315-s001.pdf]

Supplementary Online Content

Jain NB, Peterson E, Ayers GD, Song A, Kuhn JE. US geographical variation in rates of shoulder and knee arthroscopy and association with orthopedist density. *JAMA Netw Open*. 2019;2(12):e1917315. doi:10.1001/jamanetworkopen.2019.17315

eTable. CPT Codes Used for Shoulder and Knee Arthroscopy Procedures
eFigure 1. Trends in Rates of Ambulatory Knee Arthroscopy, Shoulder Arthroscopy, and Arthroscopic Rotator Cuff Repair in Select US States (2006-2016)
eFigure 2. Age and Sex–Standardized Rates of Arthroscopy in Select US States (2006-2016)
eFigure 3. Income-Standardized Rates of Arthroscopy in Select US States (2006-2016)
eFigure 4. Association of Orthopedic Surgeon Density With Rates of Arthroscopic Procedures in Select US States (2006-2016)

This supplementary material has been provided by the authors to give readers additional information about their work.

© 2019 Jain NB et al. *JAMA Network Open.*

eTable: CPT® Codes Used for Shoulder and Knee Arthroscopy Procedures

Procedure	CPT® Code	CPT® Code	CPT® Code	CPT® Code	CPT® Code	CPT® Code	CPT® Code	CPT® Code	CPT® Code	CPT® Code	CPT® Code
**Shoulder Arthroscopy**											
Rotator cuff repair	29827										
SLAP repair	29807										
Capsulorrhaphy	29806										
Subacromial decompression (with Partial acromioplasty, with coracoacromial ligament release, when performed)	29826										
Claviculectomy (Mumford)	29824										
Debridement (limited or extensive)	29822	29823									
Other shoulder arthroscopy	29805	29819	29820	29821	29825	29828					
**Knee Arthroscopy**											
Meniscectomy/Meniscal repair	29880	29881	29882	29883							
Anterior cruciate ligament/posterior cruciate ligament repair or augmentation or reconstruction	29888	29889									
Abrasion arthroplasty/microfracture	29879										
Chondroplasty	29877										
Synovectomy	29875	29876									
Other knee arthroscopy	29866	29867	29868	29870	29871	29873	29874	29884	29885	29886	29887
Excludes 29850, 29851, 29855, and 29856											
Excludes 29999 (unlisted arthroscopy)											

© 2019 Jain NB et al. *JAMA Network Open.*

eFigure 1: Trends in rates of ambulatory knee arthroscopy, shoulder arthroscopy, and arthroscopic rotator cuff repair in select U.S. states (2006-2016)

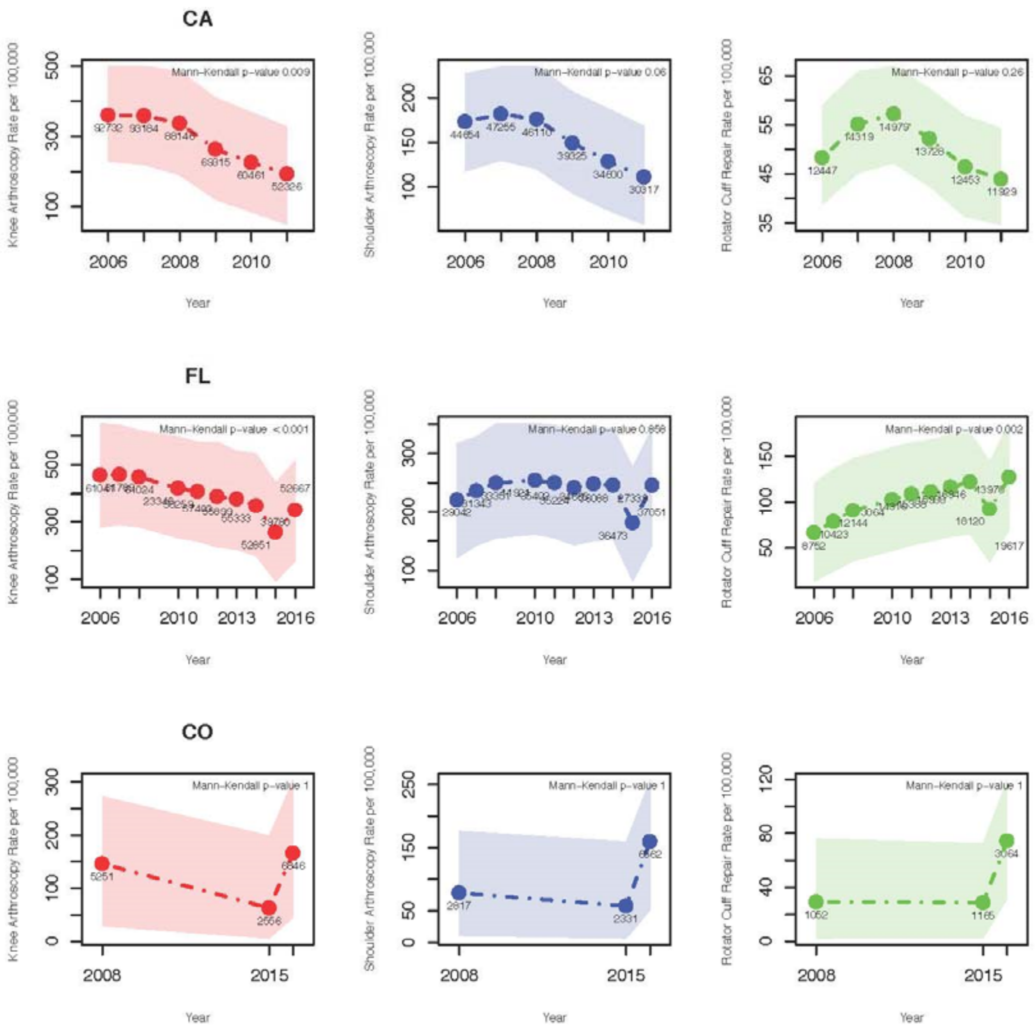

© 2019 Jain NB et al. *JAMA Network Open.*

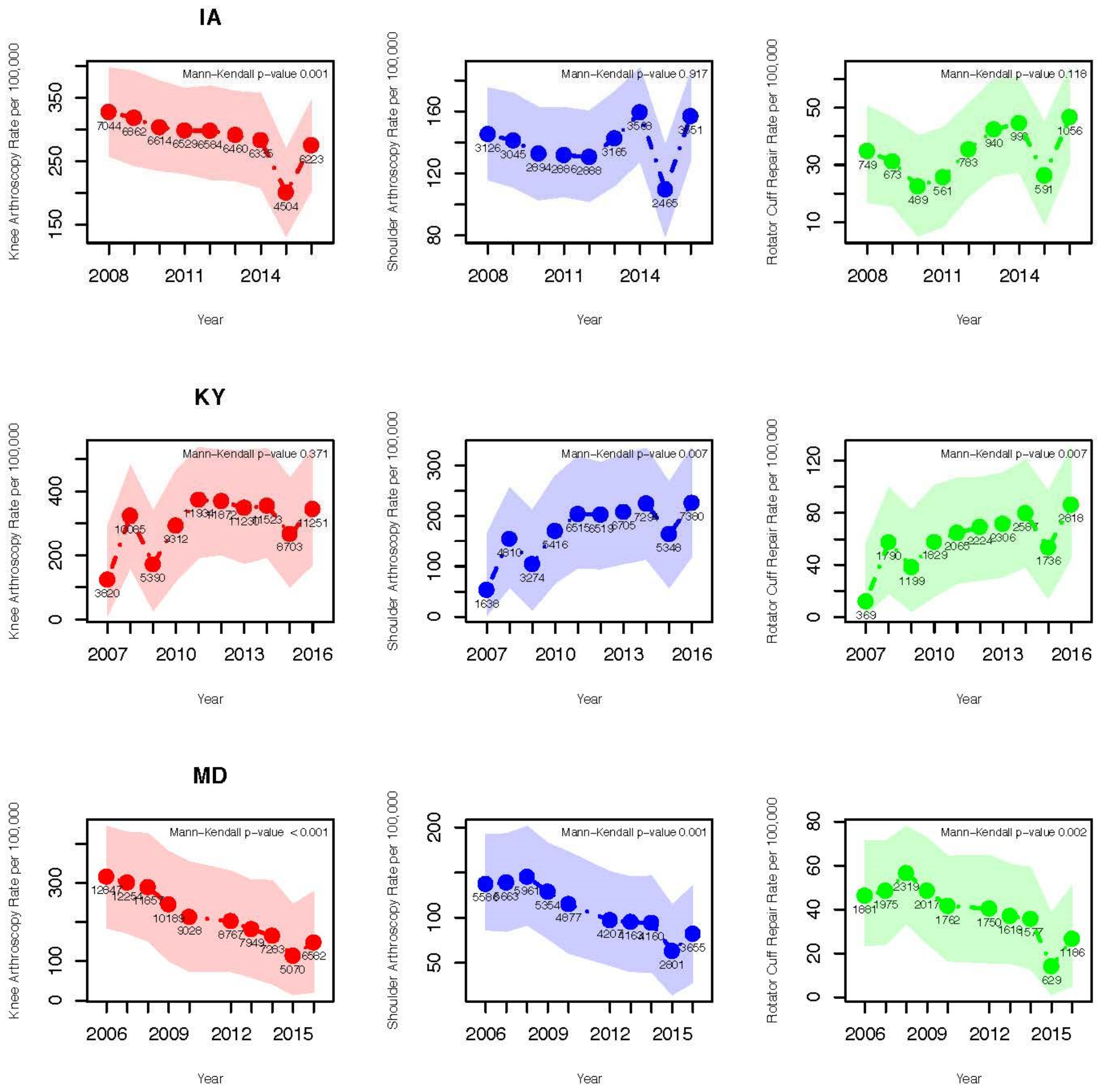

© 2019 Jain NB et al. *JAMA Network Open.*

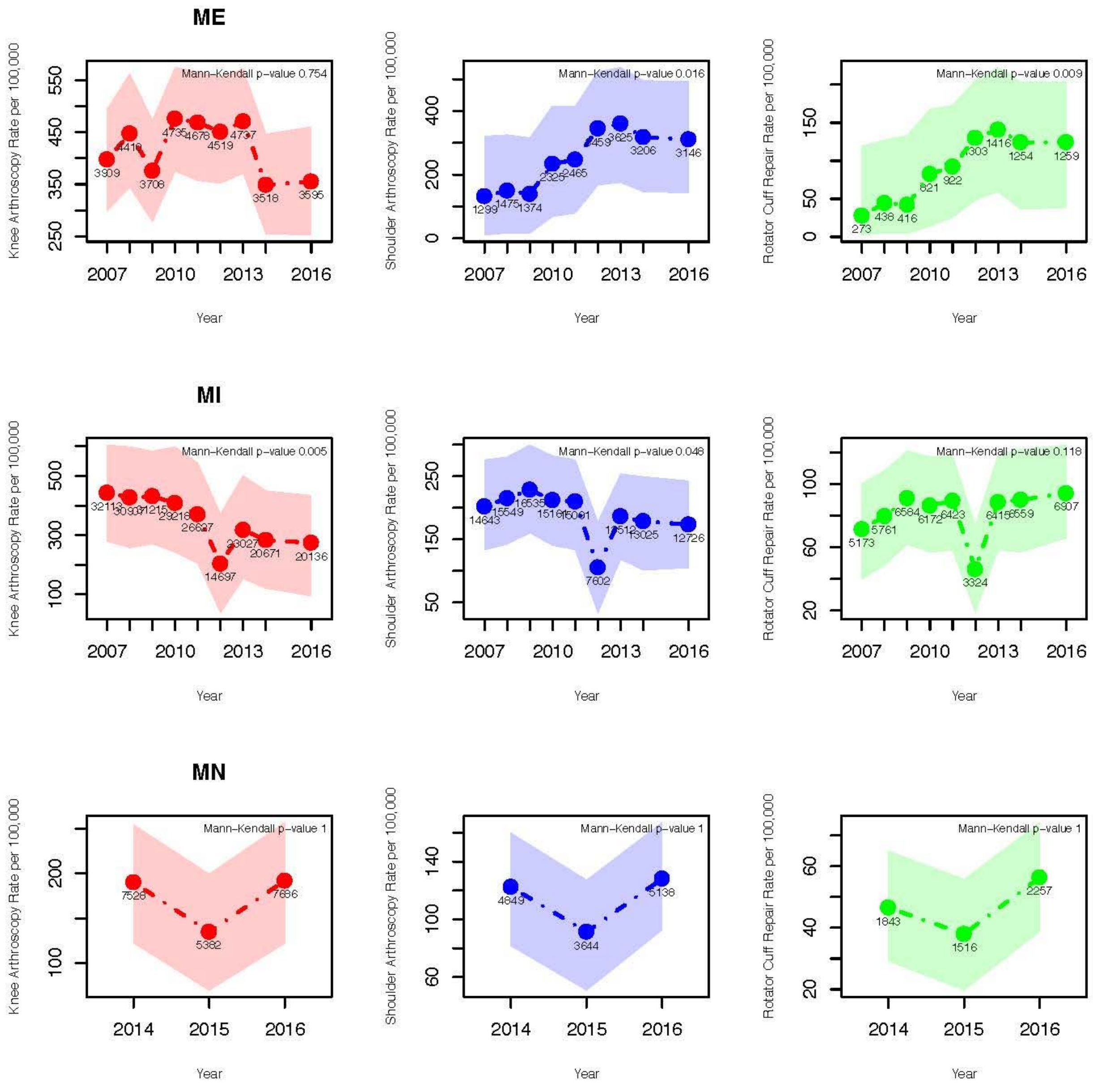

© 2019 Jain NB et al. *JAMA Network Open.*

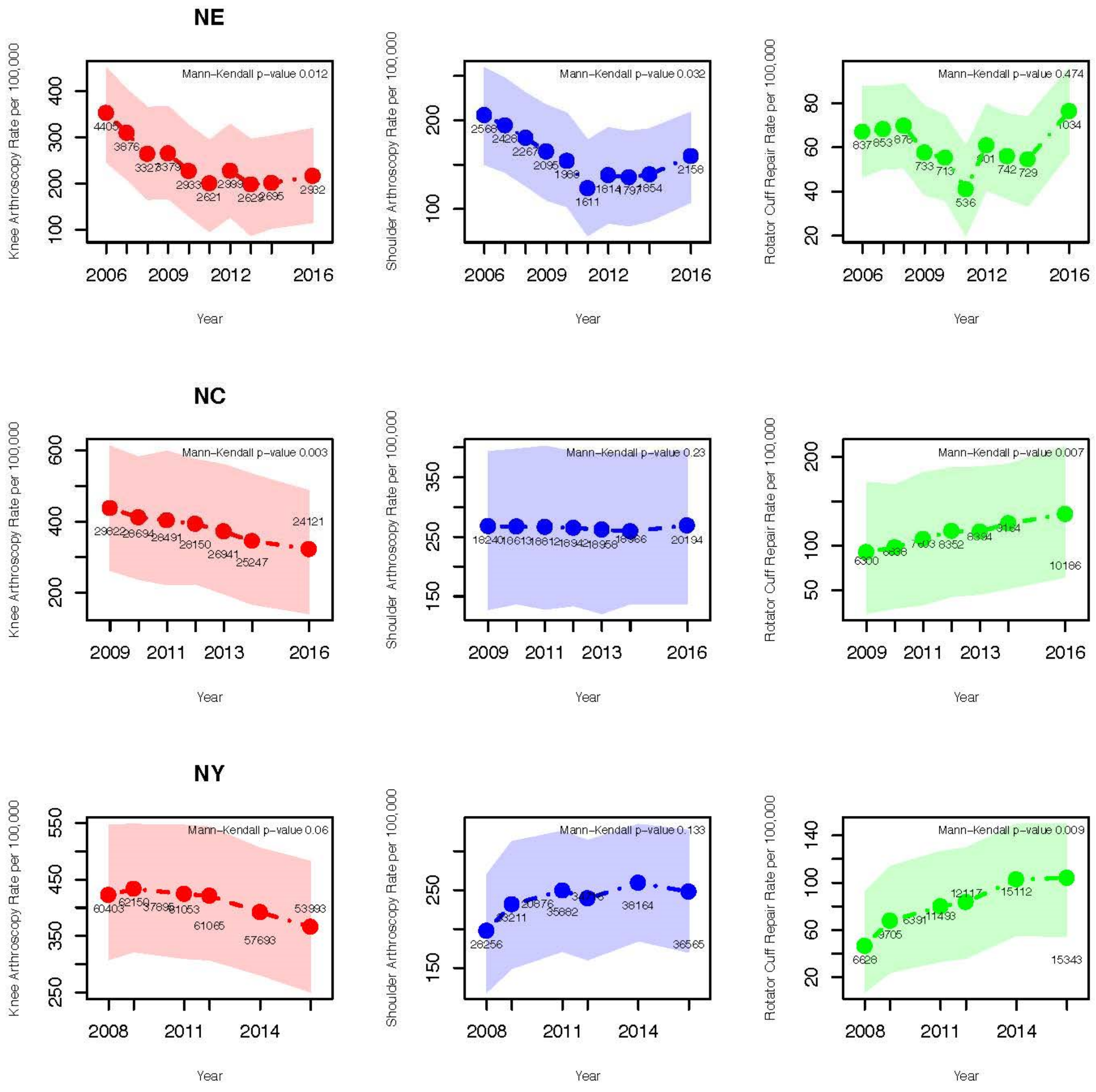

© 2019 Jain NB et al. *JAMA Network Open.*

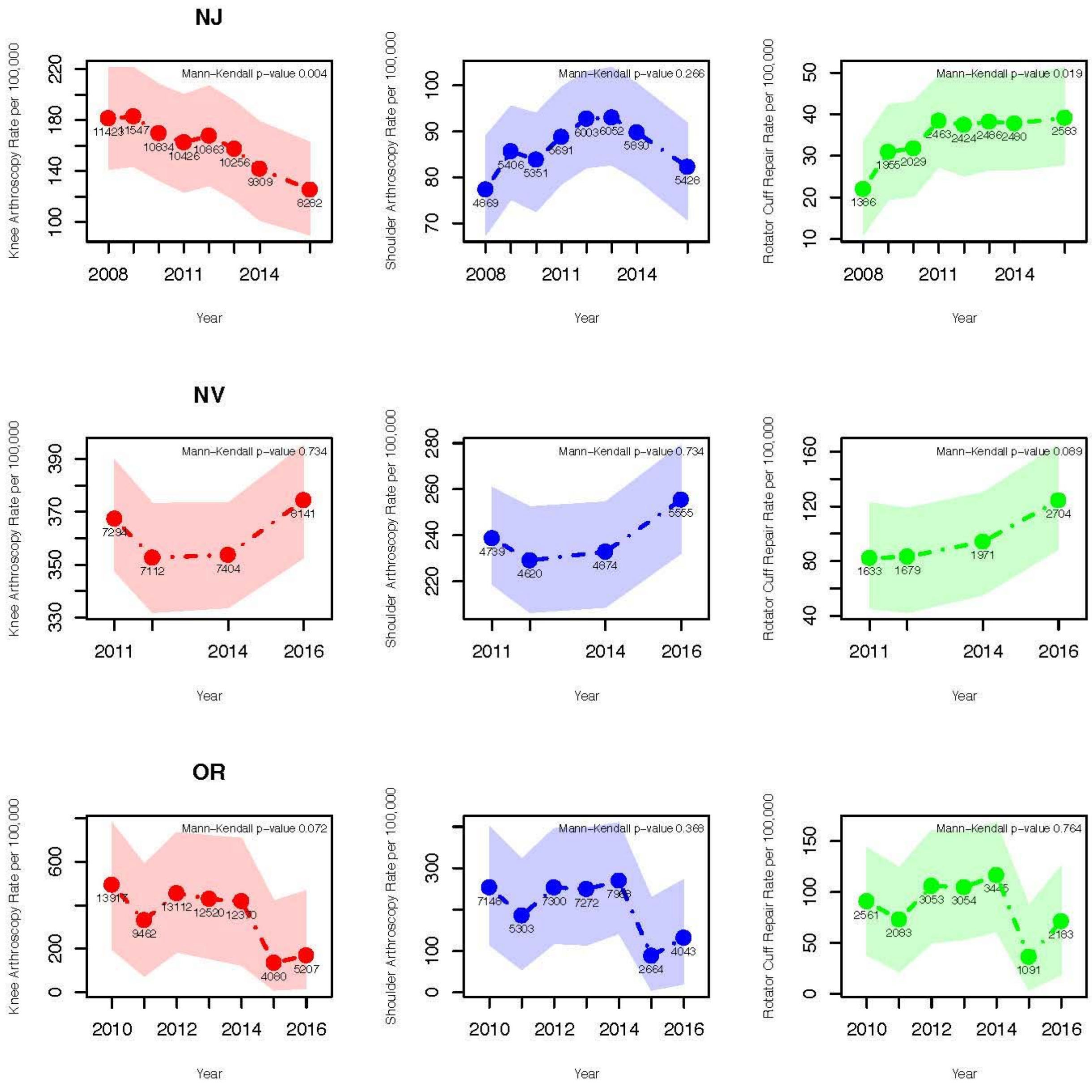

© 2019 Jain NB et al. *JAMA Network Open.*

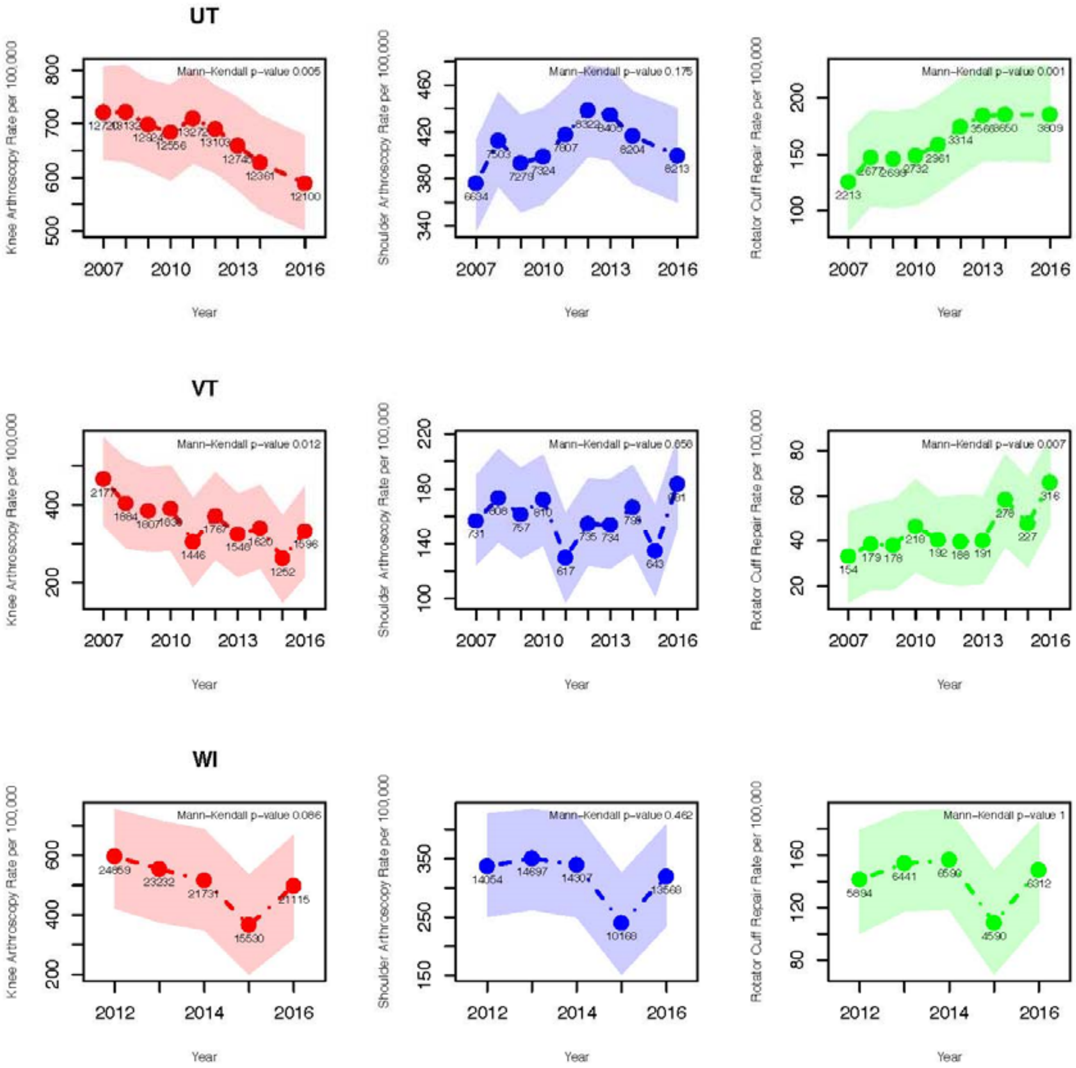

Note: Data for the following states and years was not included due to unreliable estimates: CO (2007, 2009-2014), FL (2009), IA (2007), KY (2016), MD (2011), NJ(2007), NV(2013), NY(2010, 2013), NC(2007, 2008, 2010)

© 2019 Jain NB et al. *JAMA Network Open.*

eFigure 2. Age and Sex–Standardized Rates of Arthroscopy in Select U.S. States (2006-2016)

eFigure 2a: Age and sex standardized rates of knee arthroscopy in select U.S. states (2006-2016)

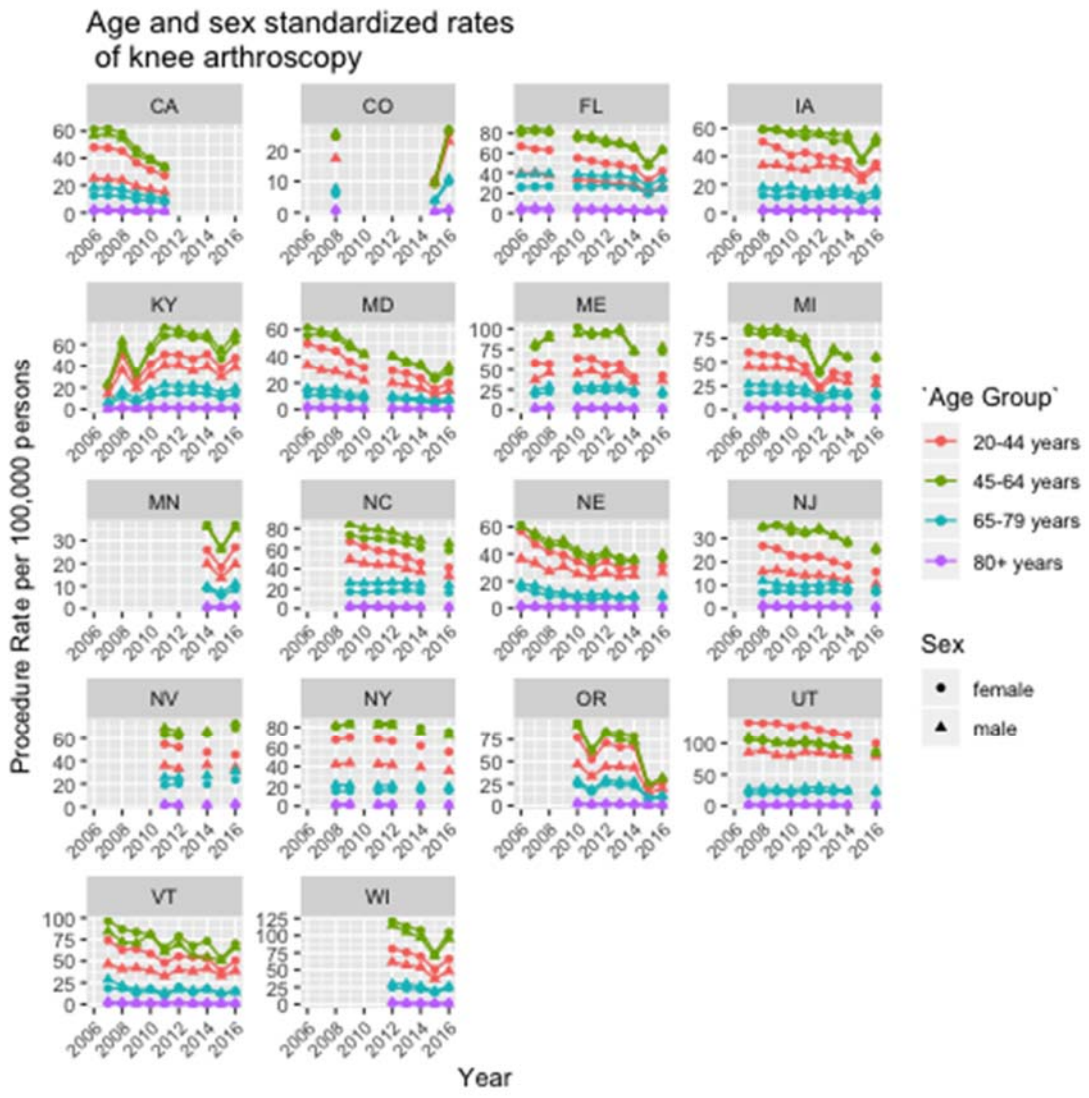

© 2019 Jain NB et al. *JAMA Network Open.*

eFigure 2b: Age and sex standardized rates of shoulder arthroscopy in select U.S. states (2006-2016)

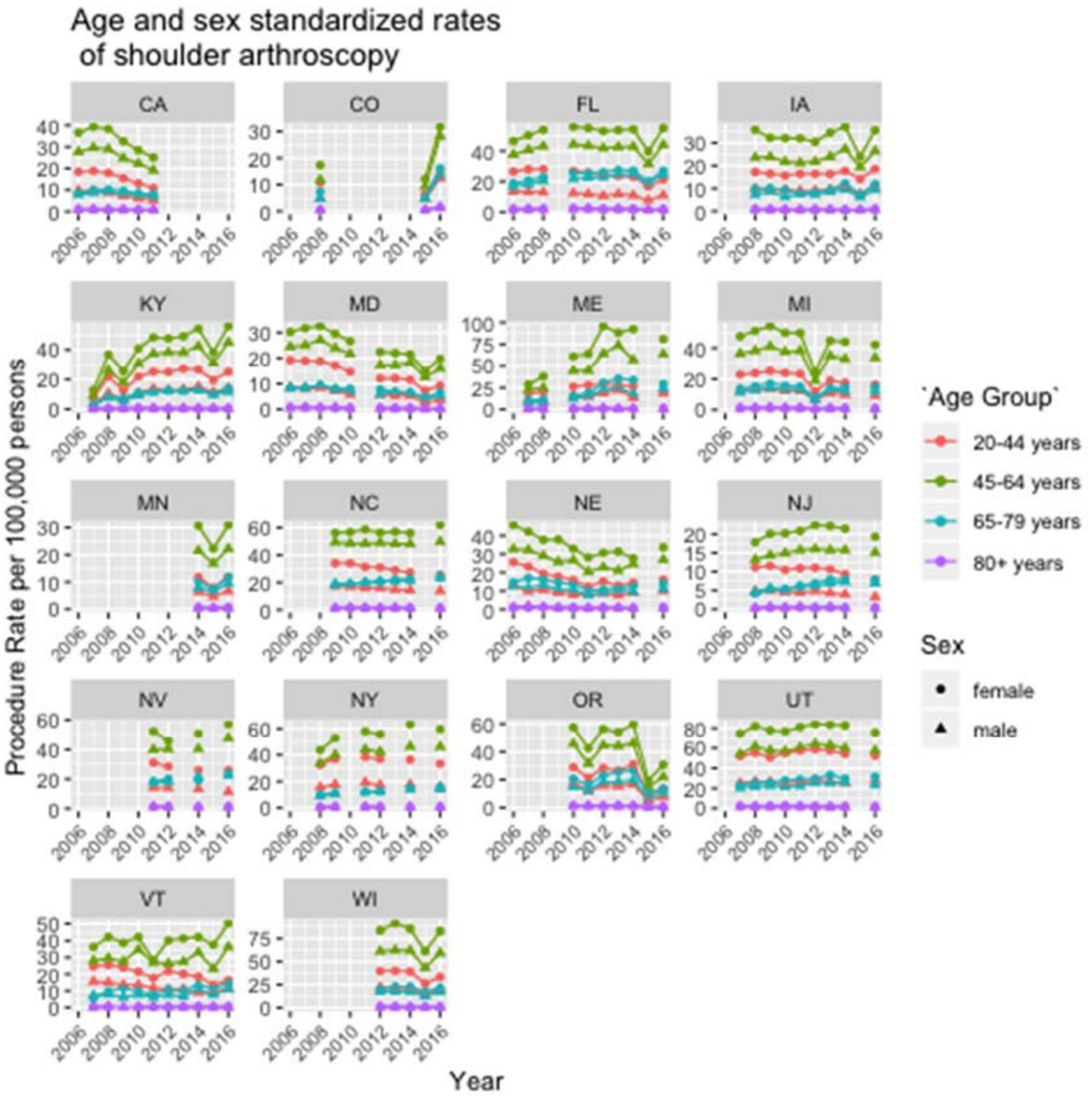

© 2019 Jain NB et al. *JAMA Network Open.*

eFigure 2c: Age and sex standardized rates of rotator cuff repair in select U.S. states (2006-2016)

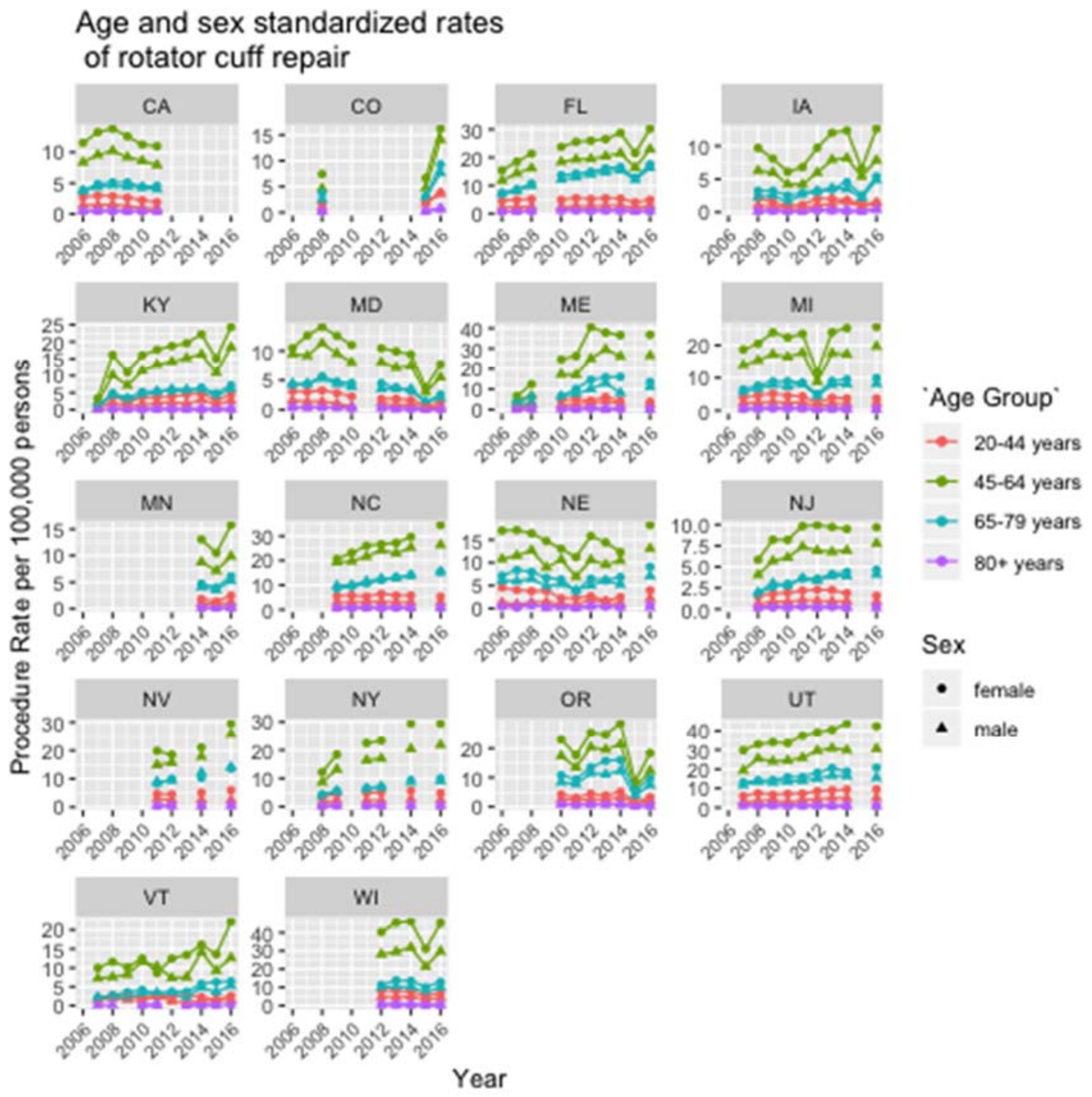

© 2019 Jain NB et al. *JAMA Network Open.*

eFigure 3. Income-Standardized Rates of Arthroscopy in Select US States (2006-2016)

eFigure 3a: Income standardized rates of knee arthroscopy in select U.S. states (2006-2016)

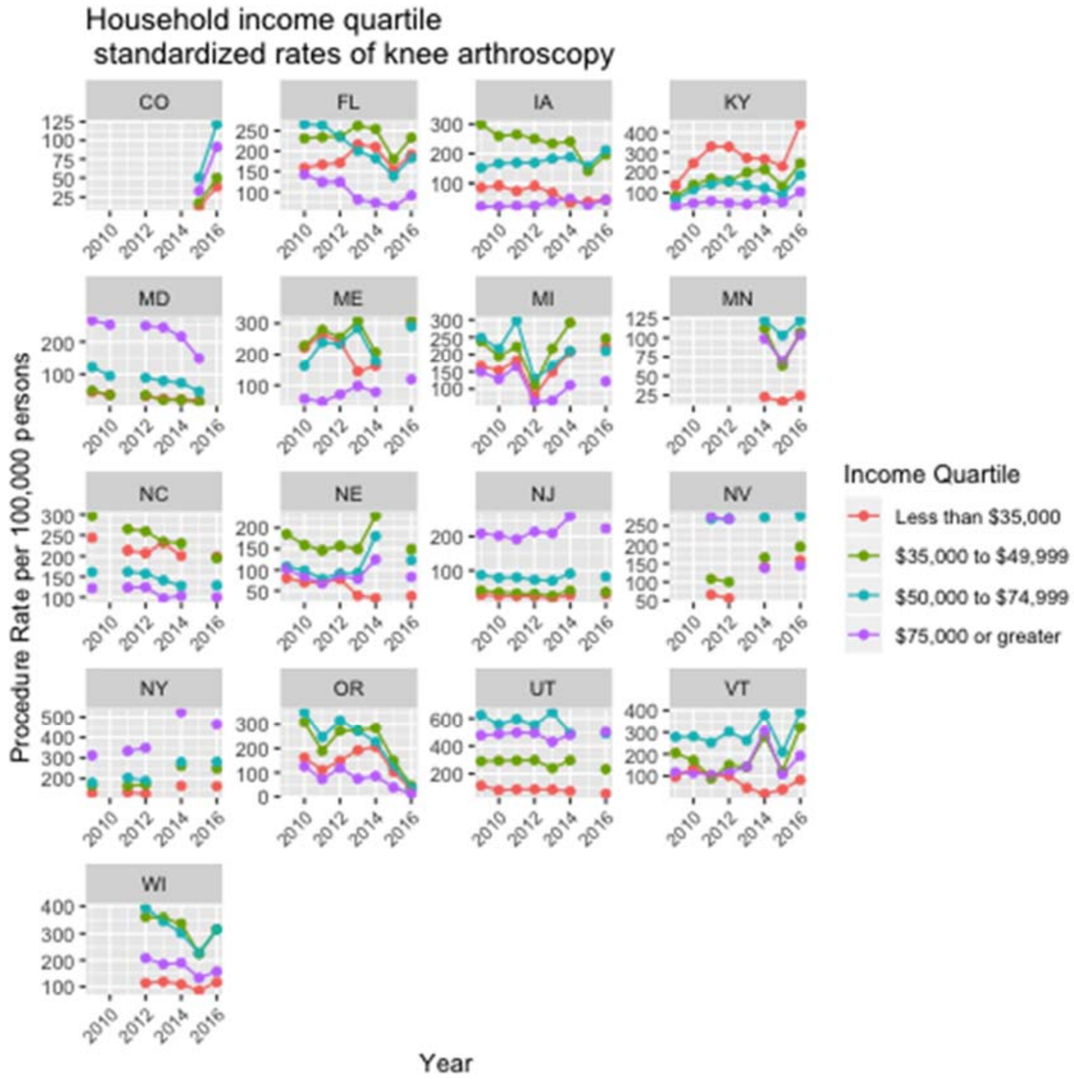

© 2019 Jain NB et al. *JAMA Network Open.*

eFigure 3b: Income standardized rates of shoulder arthroscopy in select U.S. states (2006-2016)

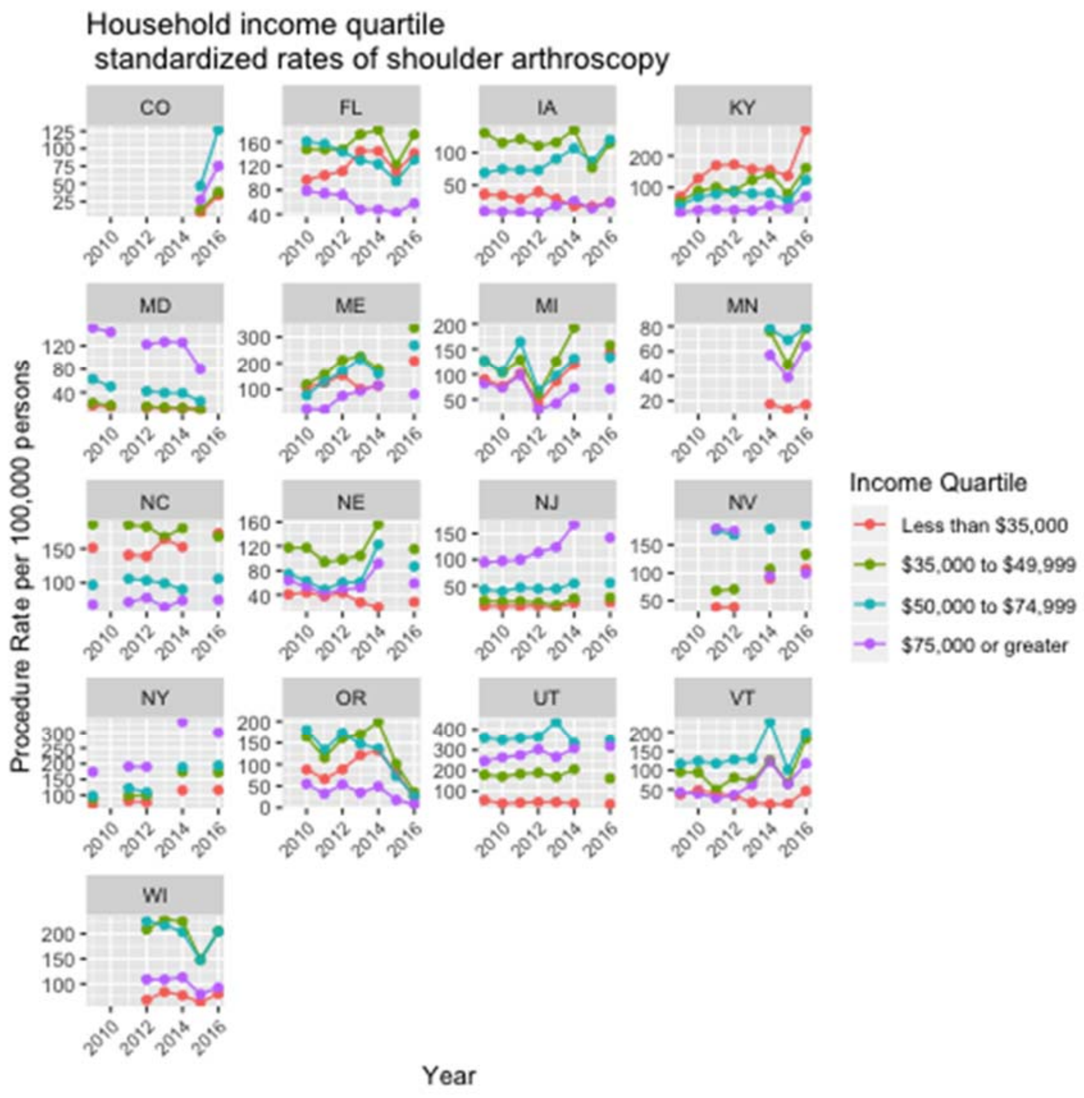

© 2019 Jain NB et al. *JAMA Network Open.*

eFigure 3c: Income standardized rates of rotator cuff repair in select U.S. states (2006-2016)

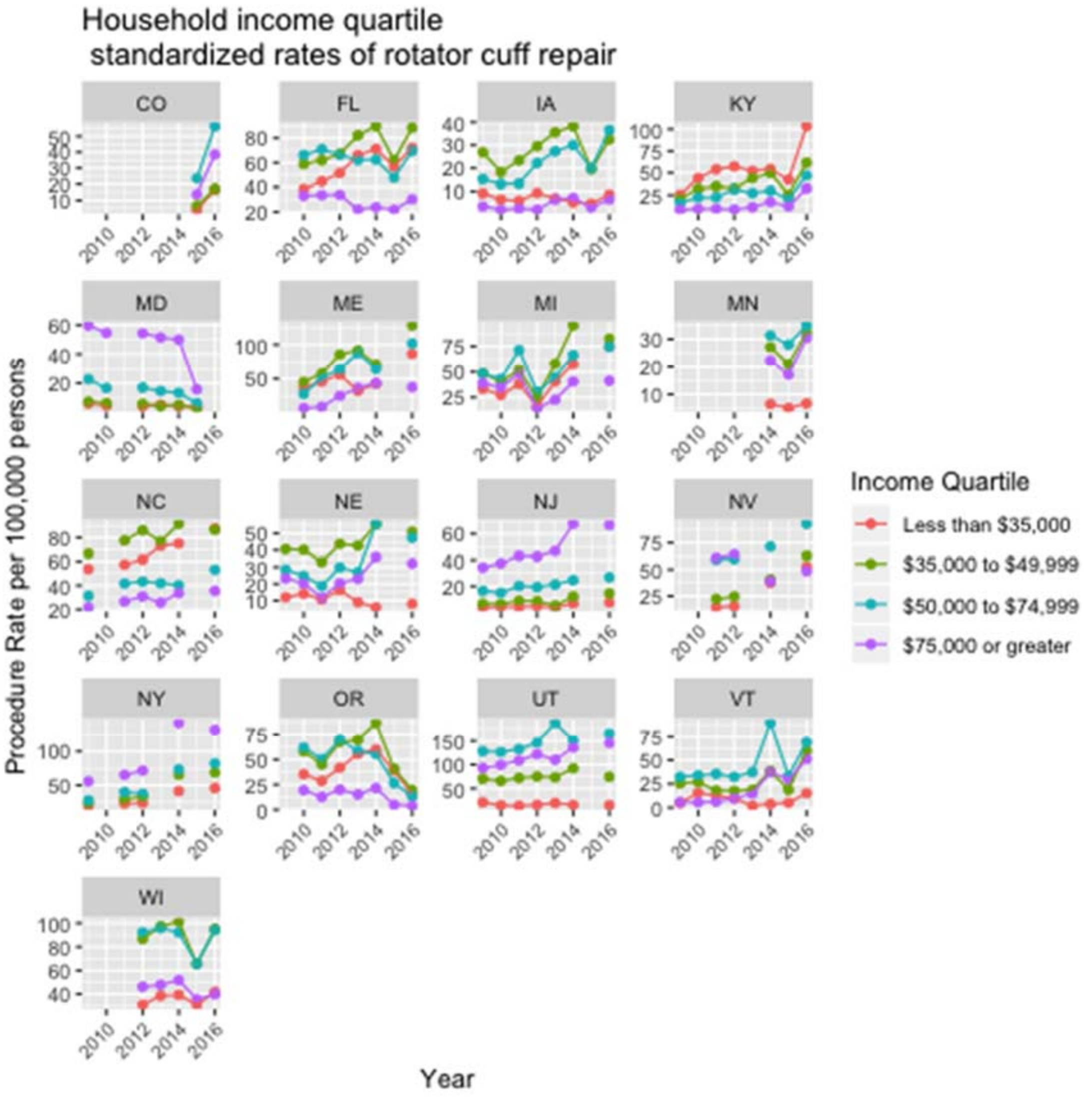

© 2019 Jain NB et al. *JAMA Network Open.*

eFigure 4. Association of Orthopedic Surgeon Density With Rates of Arthroscopic Procedures in Select US States (2006-2016)
eFigure 4a: Association of orthopedic surgeon density with procedure rates for knee arthroscopy in select U.S. states adjusting for state and time trends

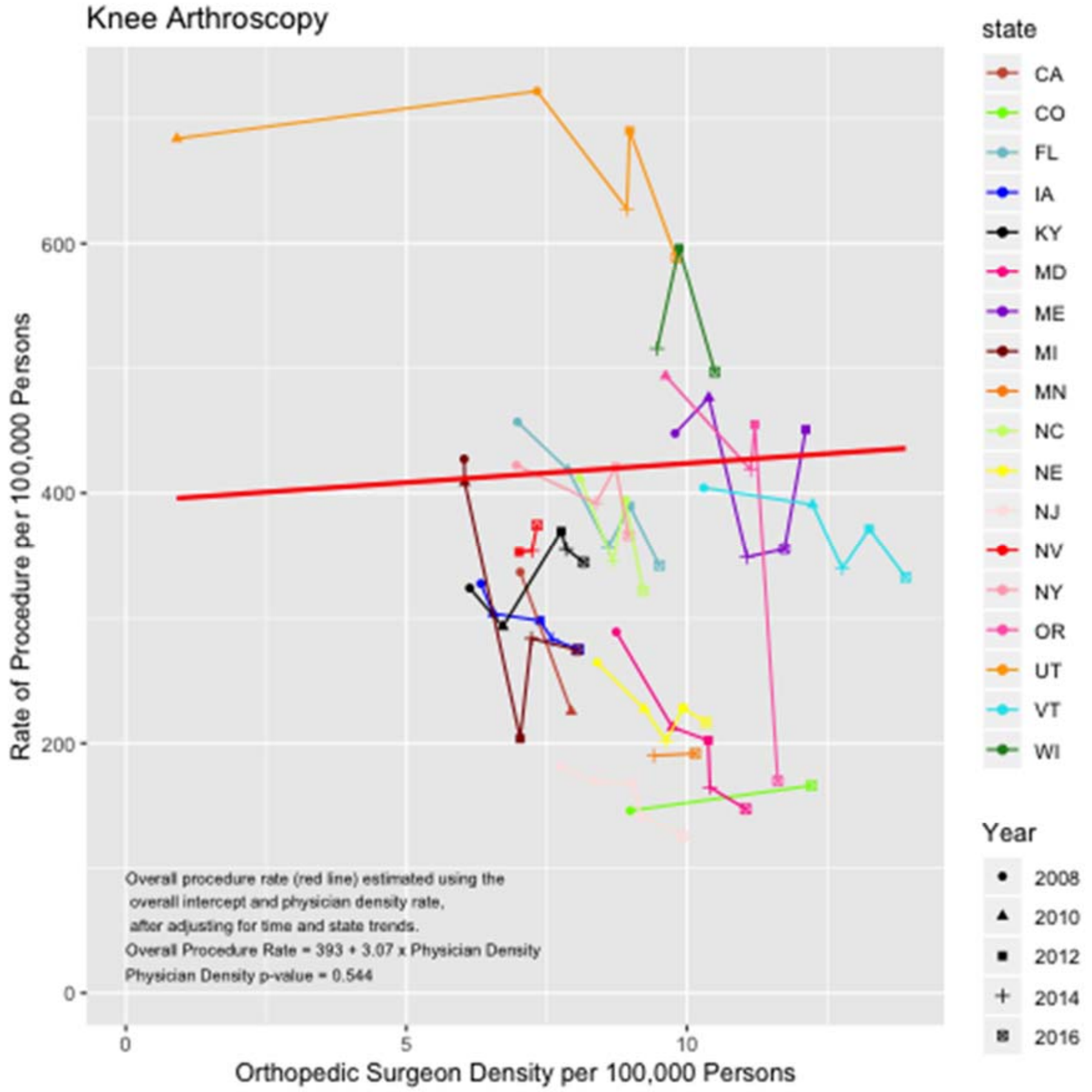

© 2019 Jain NB et al. *JAMA Network Open.*

eFigure 4b: Association of orthopedic surgeon density with procedure rates for shoulder arthroscopy in select U.S. states adjusting for state and time trends

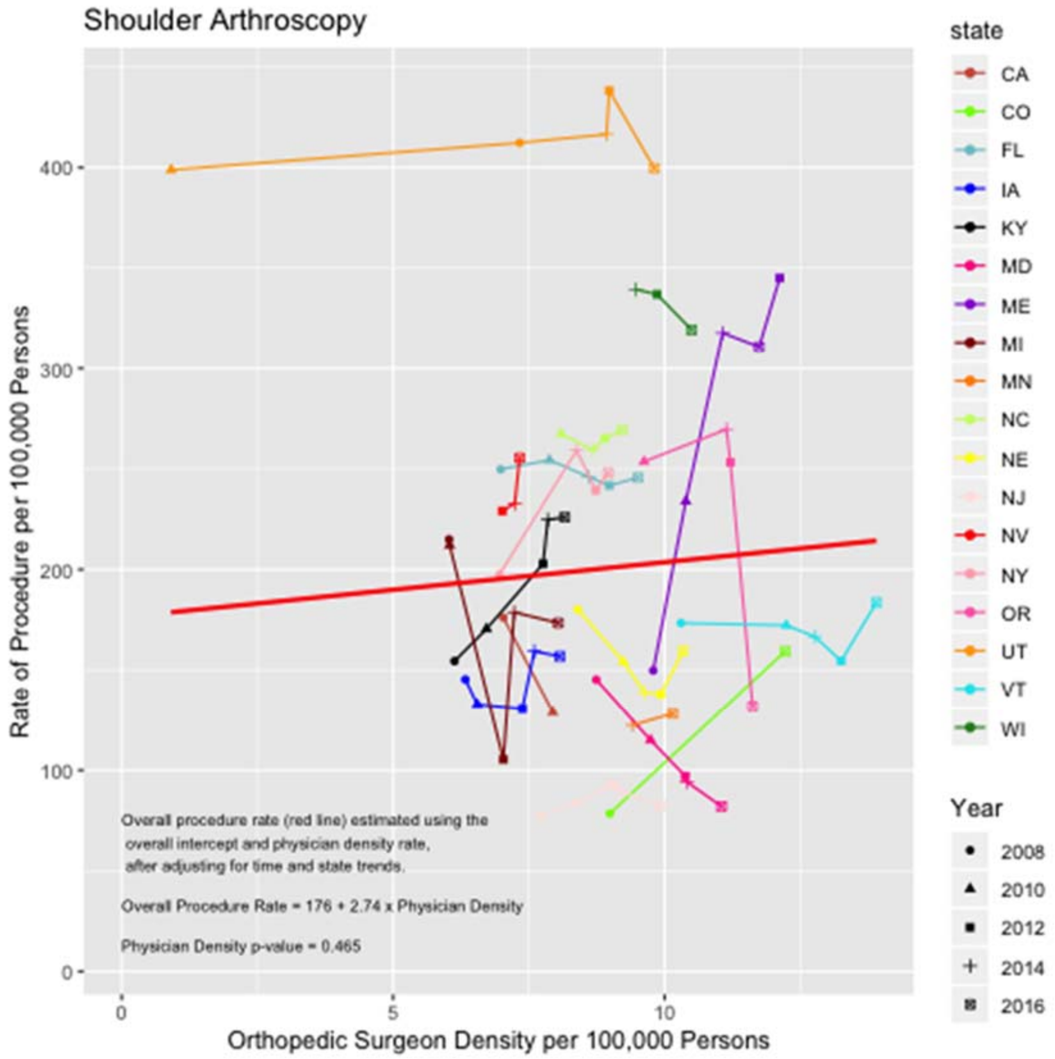

© 2019 Jain NB et al. *JAMA Network Open.*

eFigure 4c: Association of orthopedic surgeon density with procedure rates for rotator cuff repair in select U.S. states adjusting for state and time trends

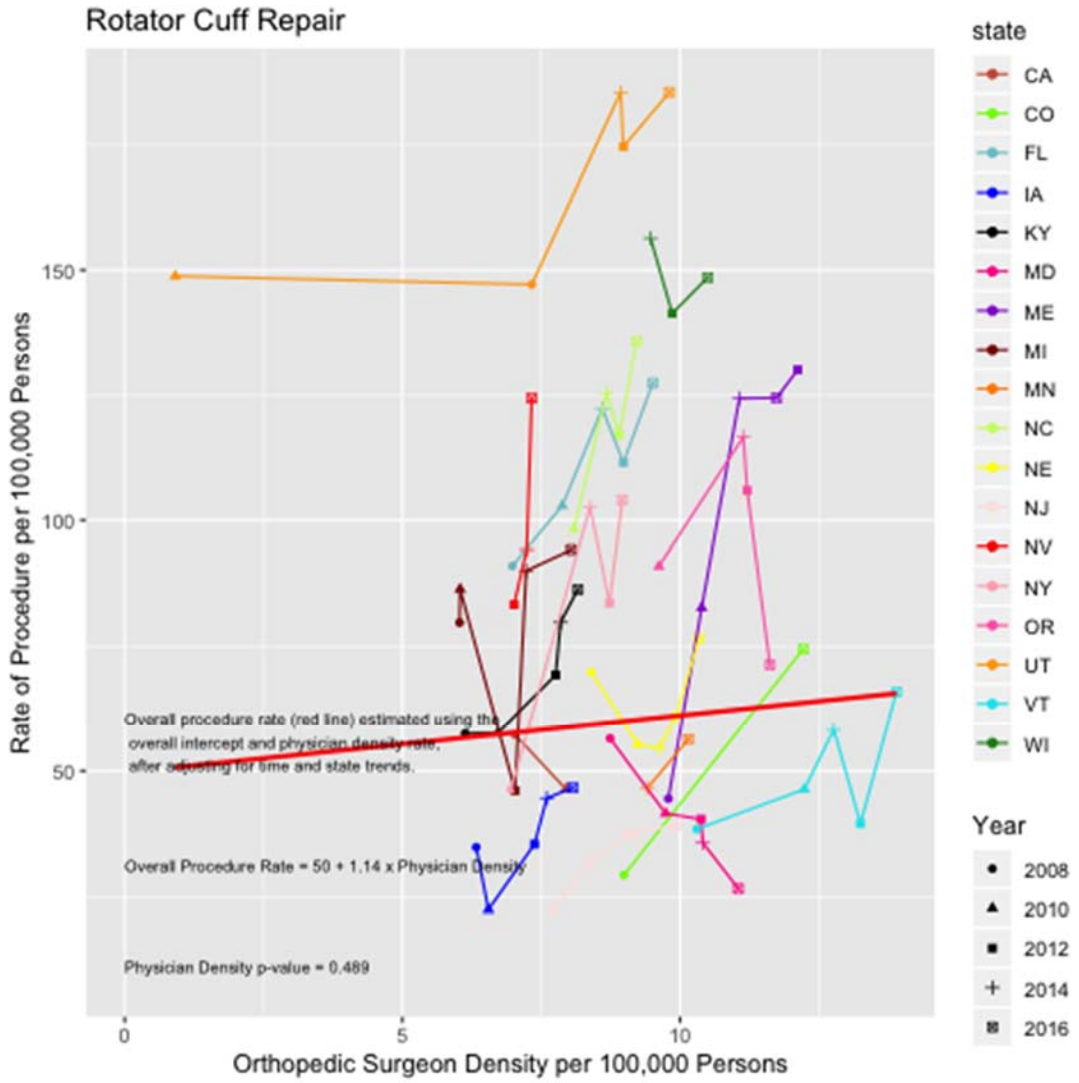

© 2019 Jain NB et al. *JAMA Network Open.*
